# Endophytes from *Ginkgo biloba* and their secondary metabolites

**DOI:** 10.1186/s13020-019-0271-8

**Published:** 2019-11-08

**Authors:** Zhihui Yuan, Yun Tian, Fulin He, Haiyan Zhou

**Affiliations:** 1 College of Bioscience and Biotechnology, Hunan Agricultural University, Changsha, 410128 China; 2 Hunan Provincial Engineering Research Center for Ginkgo Biloba, Yongzhou, 425199 China; 3 College of Chemistry and Bioengineering, Hunan University of Science and Engineering, Yongzhou, 425199 China

**Keywords:** *Ginkgo biloba*, Chinese medical plant, Endophytes, Secondary metabolites

## Abstract

*Ginkgo biloba* is a medicinal plant which contains abundant endophytes and various secondary metabolites. According to the literary about the information of endophytics from *Ginkgo biloba*, *Chaetomium*, *Aspergillus*, *Alternaria*, *Penicillium* and *Charobacter* were isolated from the root, stem, leaf, seed and bark of *G. biloba*. The endophytics could produce lots of phytochemicals like flavonoids, terpenoids, and other compounds. These compounds have antibacteria, antioxidation, anticardiovascular, anticancer, antimicrobial and some novel functions. This paper set forth the development of active extracts isolated from endophytes of *Ginkgo biloba* and will help to improve the resources of *Ginkgo biloba* to be used in a broader field.

## Background

*Ginkgo biloba* (*G. biloba*) is a deciduous tree belonging to the ginkgo genus, which is also known as *Gongsunshu*, etc. *G. biloba* is one of the most ancient plants on earth dating back more than 200 million years. Commonly *Ginkgo biloba* has been used for a medicinal plant and its seeds, leaves and fruits can be used for medicines with biological activities involving antibacteria, antioxidation, anticardiovascular and others. However, *Ginkgo* trees grow slowly and under natural conditions they need more than 20 years from planting to fruiting, which is a restricting point for its development; while its endophytics provide physiological metabolic pathways to produce numerous novel medicinal compounds which have become a hotspot [1].

The endophytics play important roles in the process of host plant growth and systematic evolution [1, 2]. During the whole life, endophytics protect their host from infectious diseases and also help to survive in adverse environment [3]. Since the unique relationships between the host plant and associated endophytes, endophytes in *G. biloba* have been recognized as important sources of a variety of novel secondary metabolites with anticancer, antimicrobial and other biological activities [4, 5].

Secondary metabolites are the chemical bank which provides a huge quantity of diverse commercial products for human medicines. First report about endophytics is that Stierle et al. isolated *Taxomyces andreanae* from phloem of *Taxus brevifolia*, which can produce taxol and related chemicals at the concentration of 24–50 ng/L [6]. From then on, more and more endophytics from pharmaceutical plants, such as *Camptotheca acuminata* [7], pine [8] and Taxus plants [9–11] were isolated. As to *G. biloba*, various endophytics including *Chaetomium*, *Aspergillus*, *Alternaria*, *Penicillium* and *Charobacter* were isolated from the root, stem, leaf, seed and bark of *G. biloba*. They produce lots of phytochemicals like flavonoids, terpenoids, and other compounds [12, 13]. 50% of these isolates showed antimicrobial activities against various pathogens. Some secondary metabolites such as 2-hexenal have been involved in the plant’s defense against pests. These bioactive metabolites are attractive to developing the commercial prodrugs and agricultural/industrial production. Most importantly, as a therapeutic drug, *G. biloba* has no side effects even after long periods of use and its phytopharmaceuticals are readily accessible throughout the world. For better using endophytic and secondary metabolites from ginkgo trees, we summarize the data previously reported.

## Endophytes in *Ginkgo biloba*

The whole plant of *G. biloba* can be used as medicine. In its root, stem, leaf, seed and bark of *Ginkgo biloba*, various endophytes have been isolated and their biological function was investigated. The conventional procedure of endophytes isolation is to wash the roots, stems or leaves of ginkgo firstly with 75% alcohol for 3 min, rinse with sterile water 3–5 times, 0.1% mercury sterilized for 2 min, rinsed with sterile water 3–5 times, cut into 0.5 cm × 0.5 cm pieces. The cutting pieces were inoculated in PDA medium at 28 °C for 4 days. After purification, ginkgo endophytes were isolated.

For the endophytic procaryotes, on the total DNA as the template, 27F(AGAGTTTGATC-CTGGGTCAG)/1492R(GGTTACCTTGTTACGACTT) as a primer, 16S rDNA was amplified. For the endophytic eukarya, ITS5 (GAAG TAAAAG TCGTAACAAGG)/ITS4 (TCCTCCGC TTA TTGA TATGC) as a primer, ITS rDNA was amplified. According to the culturing and molecular analysis between different species, the endophytics residing in *G. biloba* belong to *Chaetomium*, *Aspergillus*, *Alternaria*, *Penicillium*, *Charobacter*, etc.

### Endophytic procaryotes in *Ginkgo biloba*

**Fig. 1:**
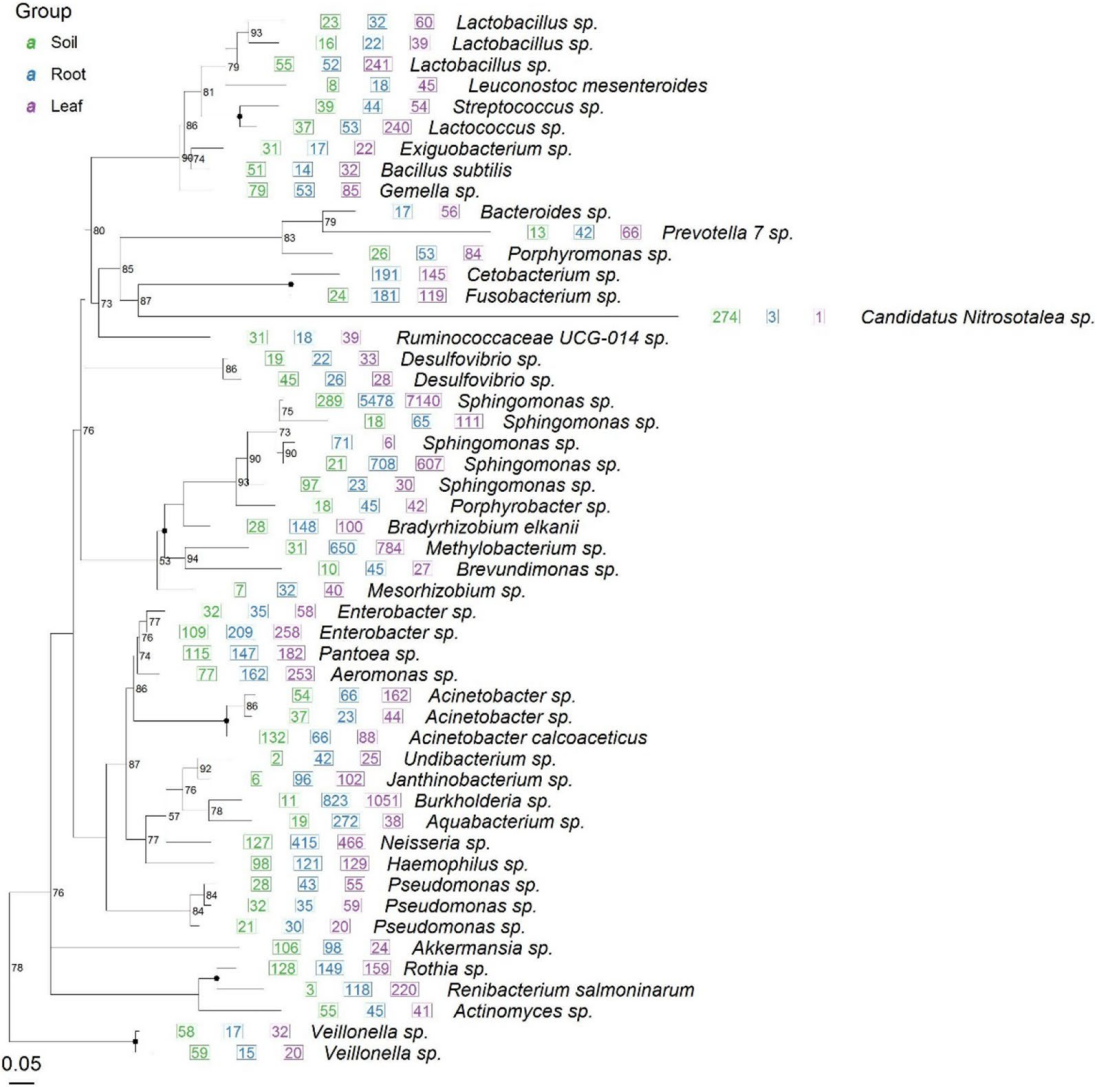
The phylogenetic tree of endophytic procaryotes from soil, root and leaf of *Ginkgo biloba*. 50 most abundant OTUs are used for display. If a number appears before the species name, it represents the total number of sequences of this OTU. If it is a graph, the graph size represents the relative abundance (percentage), and the black dot on the branch represents the bootstrap confidence greater than 95%

**Fig. 2:**
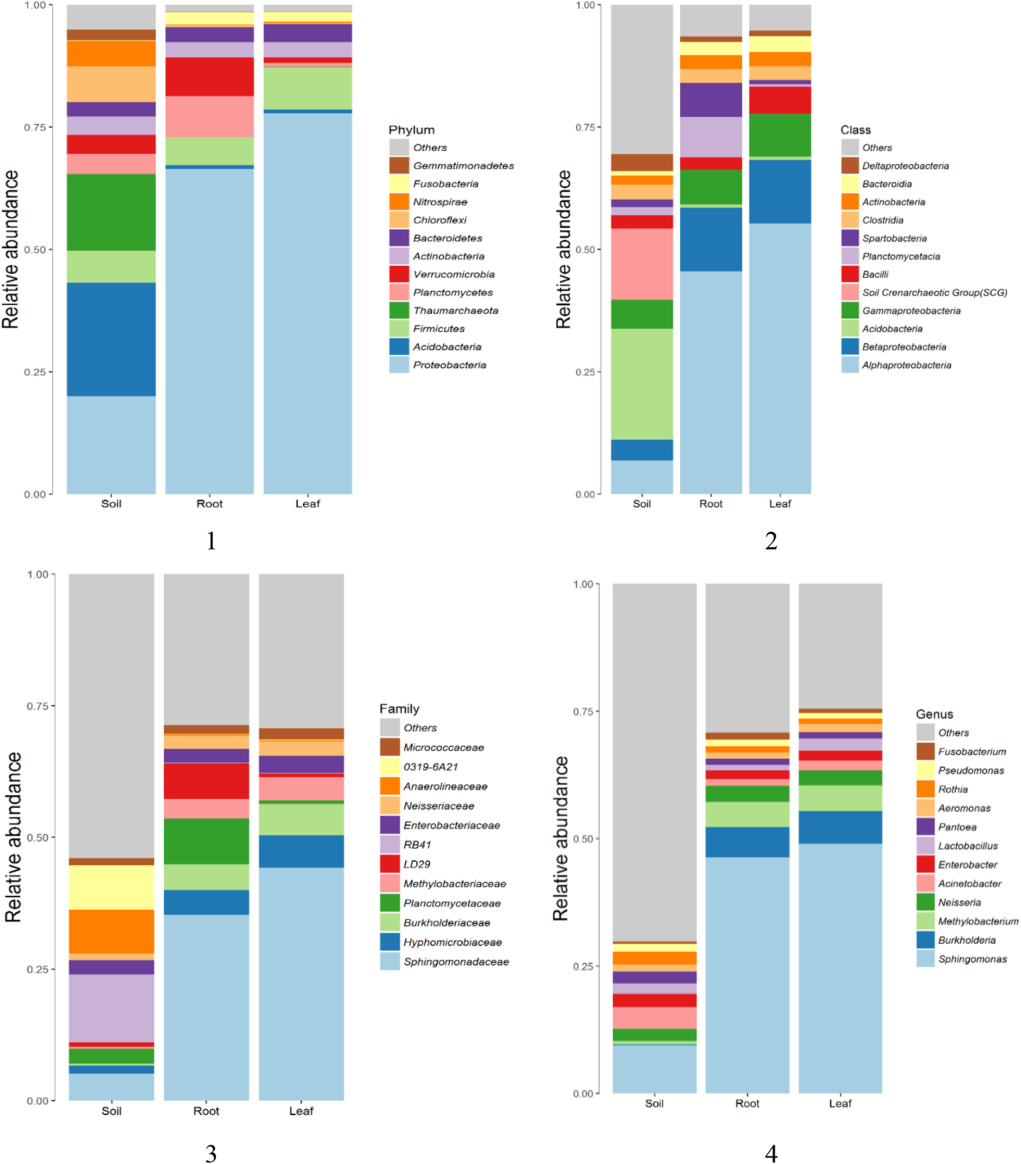
The community structure at different taxonomic levels. (1) The community structure at different phylums; (2) the community structure at different classes; (3) the community structure at different families; (4) the community structure at different genus. The percentage in parentheses indicates that only the group with the average abundance greater than this ratio is listed. All other groups are classified in others

From the previous reports, around 50 species of endophytic procaryotes were found including *Bacillus subtilis*, *Lactobacillus* sp., *Fusobacterium* sp., *Gemella* sp., *Neisseria* sp., *Pseudomonas* sp., *Rothia* sp., *Veillonella* sp., etc. Basing on 16S RNA sequence of endophytic procaryotes from previous literatures, the phylogenetic tree was constructed in Fig. 1. Amongst these procaryotes, the community structure or compositional differences at different taxonomic levels was presented in Fig. 2.

*Sphingomonadaceae* are a family of the *Alphaproteobacteria* and most abundant in *G. biloba*. An important feature is the presence of sphingolipids in the outer membrane of the cell wall [14]. In this family, some species are phototrophic which may have high nutritional value. The phototrophic bacteria are rich in amino acids, folic acid and vitamins, especially vitamin B12, biotin and coenzyme Q. Some other species are known as the ability to degrade some aromatic compounds which has the interests for environmental remediation [11].

Other abundant species are family *Hyphomicrobiaceae*, *Burkholderiaceae*, *Methylobacteriaceae*, *Enterobacteriaceae*, *Neisseriaceae* and *Micrococcaceae*. The family *Hyphomicrobiaceae* is affiliated with *Alphaproteobacteria* and members of this family are distributed everywhere in soils, freshwater, and also under the marine. This family is highly diverse morphologically and physiologically. Most are aerobic chemoheterotrophs and a few can grow anaerobically by denitrification or mixed-acid fermentation.

The *Methylobacteriaceae* comprises a large family of *Alphaproteobacteria* and contains three genera including *Methylobacterium*, *Microvirga*, and *Meganema*. *Methylobacterium* species are ubiquitous in the natural environment. Some species induce plant leaf and root nodule formation, and can promote plant growth by production of auxins [15]. Most of *Methylobacterium* are methylotrophs and they can use methanol or other one-carbon compounds as energy sources to produce proteins [16]. Otherwise, in *Methylobacterium*, common fatty acids were contained especially ubiquinone Q-10, a popular dietary supplement.

Family *Enterobacteriaceae* contains a large number of genera that are biochemically and genetically related to one another. Many of them are pathogens, such as *Salmonella*, *Shigella* or *Yersinia*, because they produce endotoxins. Endotoxins reside in the cell wall and when the cell dies and the cell wall disintegrates, endotoxins are released [9].

Family *Burkholderiaceae* belongs to the order *Burkholderiales* within the class *Betaproteobacteria*. This family is characterized by the presence of ecologically extremely diverse organisms and contains truly environmental saprophytic organisms, phytopathogens, opportunistic pathogens, as well as primary pathogens for humans and animals.

Family *Neisseriaceae* and *Micrococcaceae* are widespread in soil, subterranean cave silts, sea, glacier silts, sewage, water sludge, aerial surfaces of plants, vegetables, and various animal species and are even more distantly related to the human pathogens.

### Endophytic eukarya in *Ginkgo biloba*

**Fig. 3:**
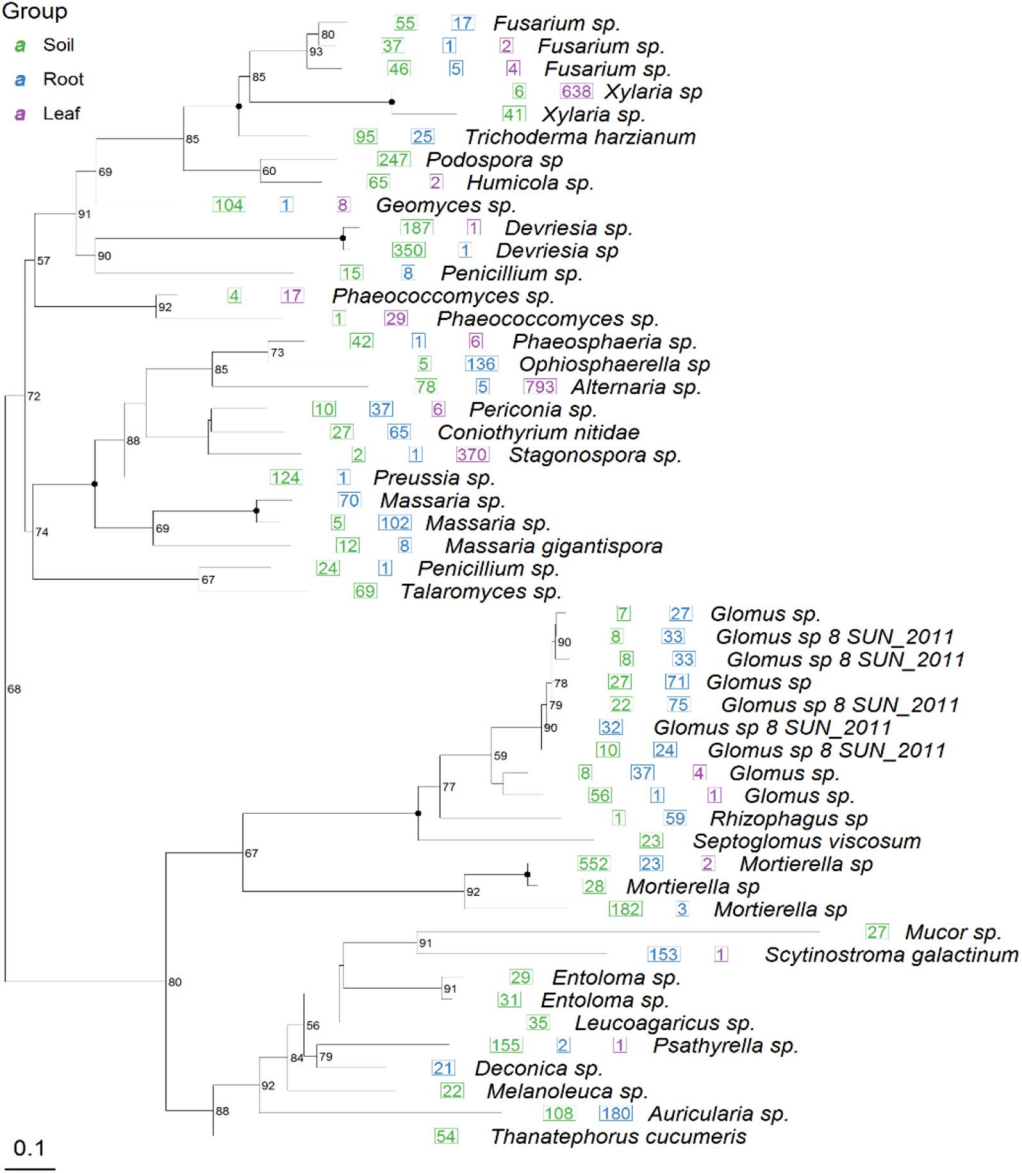
The phylogenetic tree of endophytic eukarya from soil, root and leaf of *Ginkgo biloba*. 50 most abundant OTUs are used for display. If a number appears before the species name, it represents the total number of sequences of this OTU. If it is a graph, the graph size represents the relative abundance (percentage), and the black dot on the branch represents the bootstrap confidence greater than 95%

**Fig. 4:**
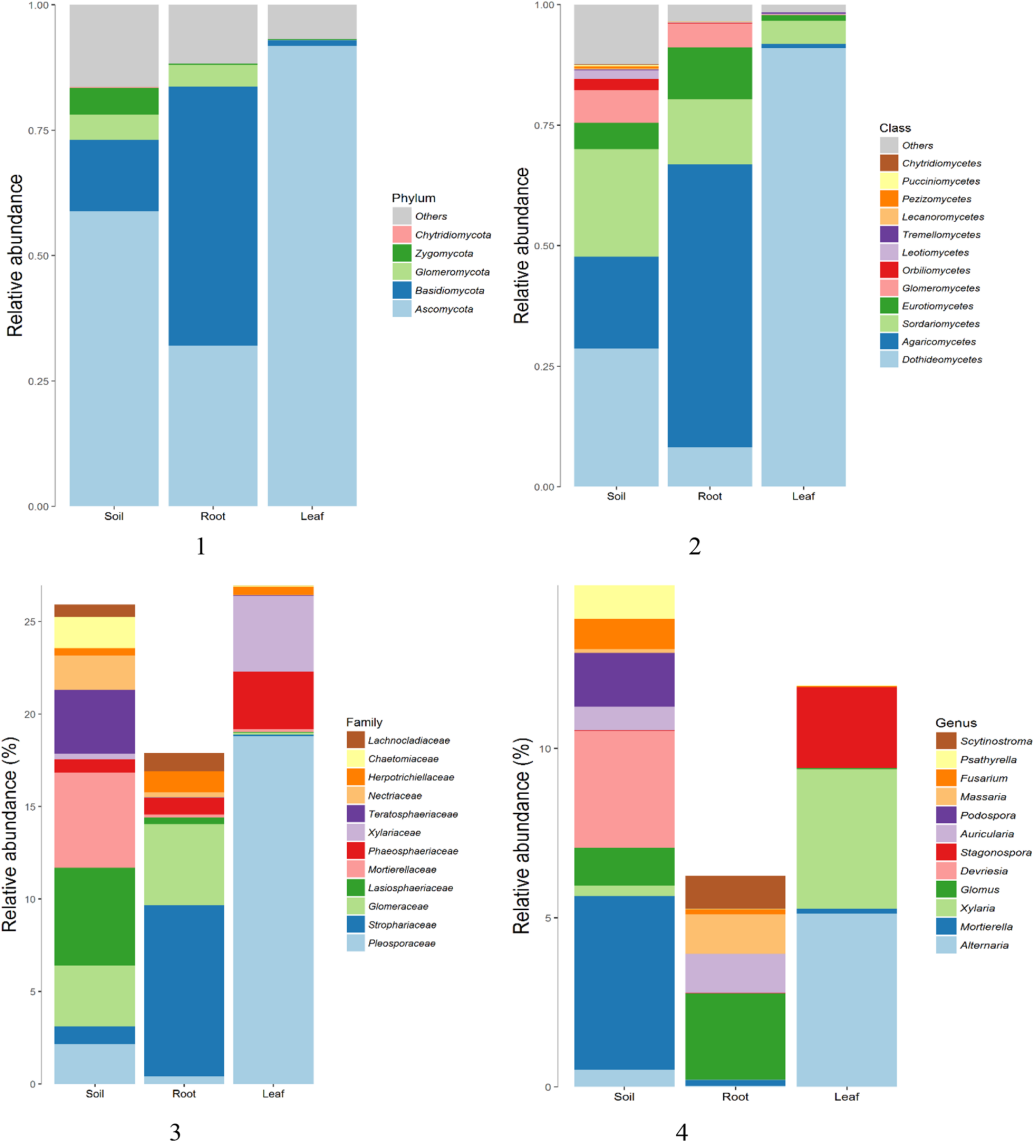
The community structure at different taxonomic levels. (1) The community structure at different phylums; (2) The community structure at different classes; (3) The community structure at different families; (4) the community structure at different genus. The percentage in parentheses indicates that only the group with the average abundance greater than this ratio is listed. All other groups are classified in others

The phylogenetic tree of endophytic eukarya (Fig. 3) was constructed basing on ITS sequence of roots and leaves of *Ginkgo biloba* from previous literatures. Amongst these endophytic eukarya, the community structure at different taxonomic levels was presented in Fig. 4.

Amongst eukarya, family *Pleosporaceae* belongs to sac fungi. The taxonomic relationship of this family to associated genera is still not determined. The classification of *Pleosporaceae* has been a challenge because of the lack of the importance of morphological characters and reference strains. From the present knowledge, the family *Pleosporaceae* includes numerous saprobic, opportunistic human and plant pathogenic taxa [17].

*Phaeosphaeriaceae* is a large and important family of fungi in the order *Pleosporales*. Species in this family have a cosmopolitan distribution, and are generally nectrotrophic or saprobic on a wide range of plants [18]. This family includes economically important plant pathogens and previously accommodated 35 sexual and asexual genera and comprised more than 300 species with a range of morphological characters [19].

The *Xylariaceae* are a family of mostly small ascomycetous fungi. It is one of the most commonly encountered groups of ascomycetes and is found throughout the temperate and tropical regions of the world. They are typically found on wood, seeds, fruits, or plant leaves, some even associated with insect nests. Most decay wood and many are plant pathogens. Phylogenetic analyses suggest that there are two main lineages in this family, Hypoxyloideae and Xylarioideae [20, 21].

## Secondary metabolites of endophytics in *Ginkgo biloba*

**Fig. 5:**
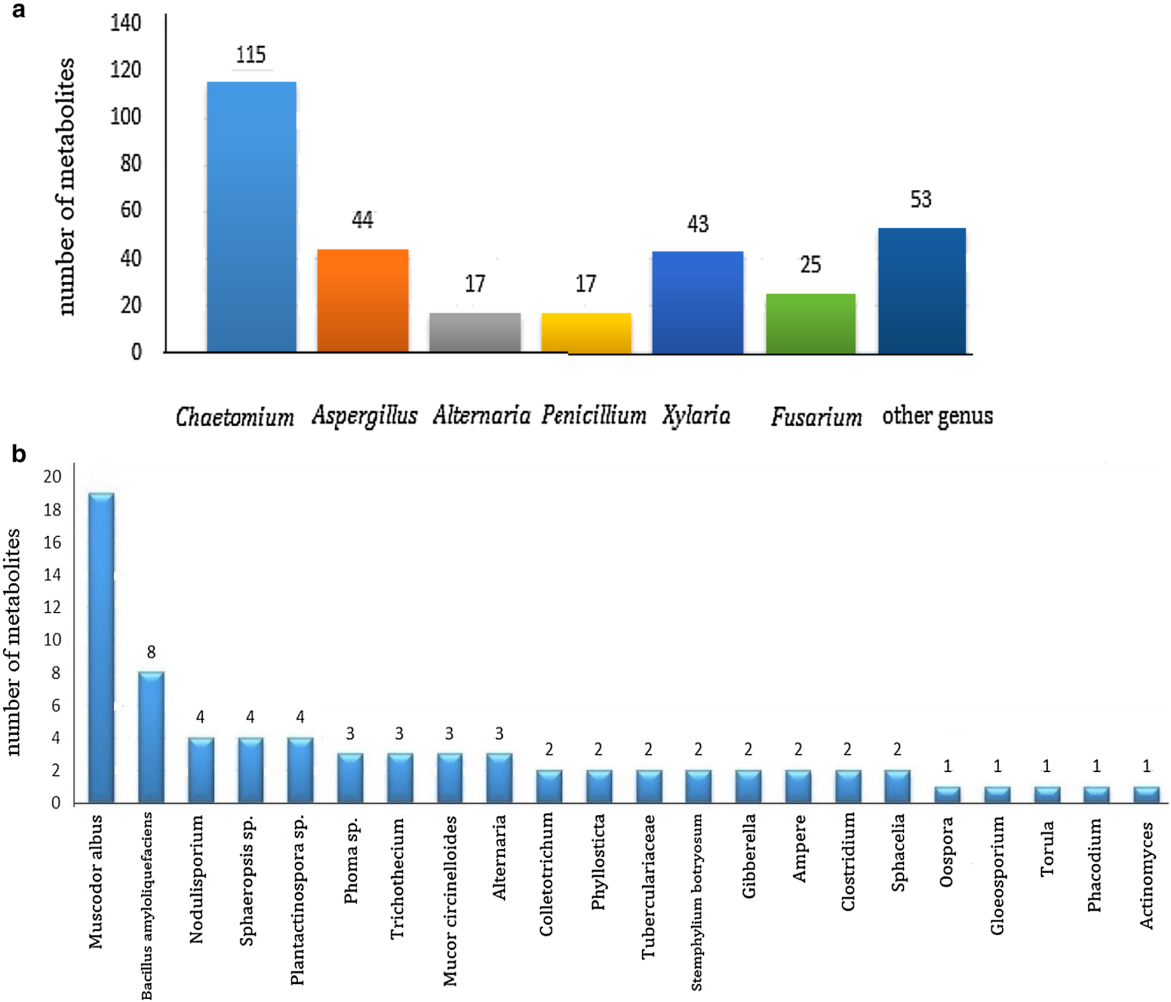
**a** The metabolite quantity of some major endophytics in *Ginkgo biloba*; **b** the metabolite quantity of some minor endophytics in *Ginkgo biloba*

A series of compounds were obtained by fermentation, extraction, and isolation from endophytics of *G. biloba*, amongst which 115 metabolites were found in the fermentation broth of *Chaetomium* fungi, 44 metabolites were found from *Aspergillus*, 43 metabolites found in the genus *Xylaria*. The amount from these three genera accounted for 72% of the secondary metabolites from endophytic procaryotes and 21% were isolated from *Fusarium*, *Alternaria* and *Penicillium*. The number of metabolites of each genus is shown in Fig. 5.

Many metabolic products from *G. biloba* have strong inhibitory effects on pathogenic bacteria *Staphylococcus aureus*, *Enterococcus faecalis*, and *Pseudomonas aeruginosa*. The secondary metabolites of *Ginkgo*, such as flavonoids and ginkgolides, are drugs or prodrugs used in the treatment of peripheral arterial diseases, neurological disorders, sclerosis of cerebral arteries, and cerebral ageing.

### Secondary metabolites of *Chaetomium*

**Fig. 6:**
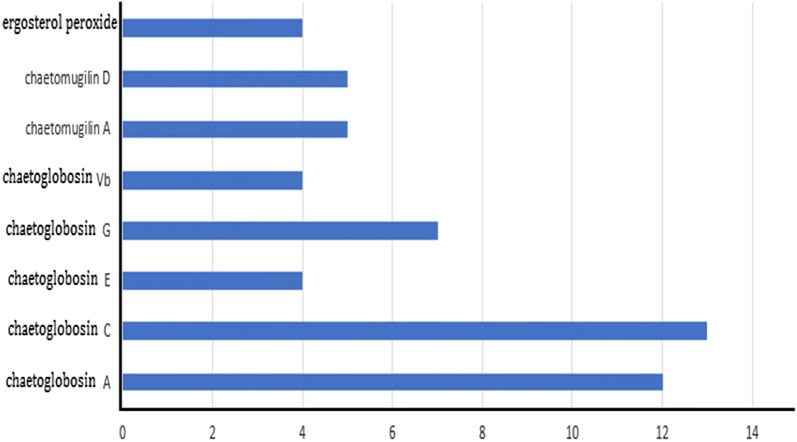
The quantity of different kinds of metabolites from *Chaetomium*

*Chaetomium* is the largest type of endophytic fungus from *G. biloba* and its secondary metabolites are biologically diverse. *Chaetomium globosum* is one of main endophytics. A total of 115 metabolites were isolated from the fermentation broth of *Chaetomium globosum* (see Fig. 6 and Table 1). Among them, chaetoglobosin A, chaetoglobosin C, chaetoglobosin E, chaetoglobosin G, chaetoglobosin Vb, chaetomugilin A, chaetomugilin D and ergosterol peroxide (peroxyergosterol; 5α, 8α-peroxy-(22E, 24R)-ergot-6,22-diene-3β-ol), which has been reported in many literatures, may be a research hotspot. Among these compounds, chaetomugilin A, chaetomugilin D, chaetoglobosin A and chaetoglobosin C have strong cytotoxic activity [22].

**Table 1:** Secondary metabolites of *Chaetomium* in *Ginkgo biloba*

No.	Metabolites	CAS number	Molecular structure	Endophytes	Application	References
1	(22E, 24R)-ergosta-7,22-diene-3β,5α,6β-triol/cerevisterol	516-37-0		*Chaetomium globosum*		[33]
2	(22E, 24R)-ergosta-7,22-diene-3β,5α,6β,9α-tetraol	88191-06-4		*Chaetomium globosum*		[44]
3	(7Z,11E)-7,11-Hexadecadien-1-yl acetate	53042-79-8		*Chaetomium globosum* No. 16	Pesticide	[45]
4	(E,E)-2,4-Decadienal	25152-84-5		*Chaetomium globosum* No. 16	Food_additive; fragrance	[45]
5	(Z)-9-Hexadecenoic acid, methyl ester	1120-25-8		*Chaetomium globosum* No. 16		[45]
6	(Z,Z)-9,12-Octadecadienoic acid	60-33-3		*Chaetomium globosum* No. 16	Biosynthesis of prostaglandins and cell membranes	[45]
7	1-(3-Acetyl-2,2-dimethylcyclopropyl)-2-methyl-1-propanone	77142-84-8		*Chaetomium globosum* T16		[49]
8	1-(3-Methoxy-2-pyrazinyl)-2-methyl-1-propanone	98618-81-6		*Chaetomium globosum* T16		[46]
9	1,3-Dioxolane, 2-methoxy	19693-75-5		*Chaetomium globosum* T16		[46]
10	1-Eicosene	3452-07-1		*Chaetomium globosum* No. 16		[45]
11	1-Trimethylsilyl methanol	3219-63-4		*Chaetomium globosum* T16		[46]
12	2,3,4-Trimethyl-5,7-dihydroxy-2,3-dihydrobenzofuran	1824584-79-3		*Chaetomium globosum*		[47]
13	2,4,5-Trimethyl-1,3-dioxolane	3299-32-9		*Chaetomium globosum* T16	Flavors	[46]
14	2,4-Decadienal	2363-88-4		*Chaetomium globosum* No.16	Food additive	[20, 21]
15	2′-*O*-Methyladenosine	2140-79-6		*Chaetomium globosum*	Inhibition of vaccinia virus growth	[47]
16	2′-Deoxyadenosine	958-09-8		*Chaetomium globosum*	Anti-tumor and antiviral nucleoside drugs (cladribine)	[44]
17	20-Dihydrochaetoglobosin A	149560-98-5		*Chaetomium globosum*		[47]
18	21 Methoxy-Chaetoglobosin F			*Chaetomium globosum*		[47]
18	2-Cyclohexyl-hex-5-en-2-ol	959261-17-7		*Chaetomium globosum* T16		[46]
19	2-Ethyl-5-propylphenol	72386-20-0		*Chaetomium globosum* T16		[46]
20	2-Methyl-5-propyl-2,4-dihydro-3H-pyrazol-3-one	31272-04-5		*Chaetomium globosum* T16		[46]
21	2-Octyl-cyclopropaneoctanal	56196-06-6		*Chaetomium globosum* No.16		[45]
22	3,4-Dihydroxyphenyl acetic acid	102-32-9		*Chaetomium globosum*	A metabolite of dopamine, Cytoplasm, Encephalitis, Hypothyroidism, Alzheimer’s disease, Colorectal cancer	[47]
23	3-Methylorsellinic acid	4707-46-4		*Chaetomium globosum* ZY-22	Neuroprotective Activity	[46]
24	4-Aminophenylacetic acid/p-aminophenylacetic acid/4-aminophenylacetic acid	1197-55-3		*Chaetomium globosum*	Anti-inflammatory Inhibition colitis	[47]
25	4-Methyl-1-hepten-5-one	26118-97-8		*Chaetomium globosum*		[46]
26	5-(hydroxymethyl)-1H-pyrrole-2-carbaldehyde	67350-50-9		*Chaetomium globosum*	Hapten, produces advanced glycation end-products (AGEs)	[47]
27	5′-Epichaetovirdin A	1308671-17-1		*Chaetomium globosum* No. 12		[45]
28	5′-Deoxy-5′-methylamino-adenosine	No cas no.		*Chaetomium globosum*		[47]
29	9(11)-dehyoergosterol peroxide	86363-50-0		*Chaetomium globosum* ZY-22		[44]
30	9,12-Octadecadien-1-ol	1577-52-2		*Chaetomium globosum* No. 16		[45]
31	Acetaldehyde, diethyl acetal	105-57-7		*Chaetomium globosum* T16	Used in fruit, rum and whisky flavour	[46]
32	Adenosine	58-61-7		*Chaetomium globosum* ZY-22	Vasodilatory, anti-arrhythmic and analgesic activities adenosine is an adenosine receptor agonist	[46]
33	Allantoin	97-59-6		*Chaetomium globosum*	Healing, soothing, and anti-irritating properties anti-acne products, sun care products, and clarifying lotions	[48]
34	alpha-Methylstyrene	98-83-9		*Chaetomium globosum*	Membrane adhesives and sealant chemicals	[48]
35	Anthranilic acid	118-92-3		*Chaetomium globosum* MX-0510	A water-soluble vitamin	[33]
36	Benzeneacetic acid	103-82-2		*Chaetomium globosum* No. 16	Used in the manufacture of penicillin and bendazol	[45]
37	Benzeneacetic acid, methyl ester	101-41-7		*Chaetomium globosum* No. 16	Used in the manufacture of atropine	[45]
38	Benzeneethanol/phenylethyl alcohol	60-12-8		*Chaetomium globosum*	Essence	[45]
39	Butyraldehyde, 4-phenyl	18328-11-5		*Chaetomium globosum* T16		[46]
40	Cerebroside B	88642-46-0		*Chaetomium globosum* ZY-22		[46]
41	Cerebroside C	98677-33-9		*Chaetomium globosum* ZY-22		[46]
42	Chaetoglobosin A	50335-03-0		*Chaetomium globosum*		[44, 49]
43	Chaetoglobosin B	50335-04-1		*Chaetomium globosum* CDW7		[48]
44	Chaetoglobosin C	50645-76-6		*Chaetomium globosum*		[26, 28]
45	Chaetoglobosin D	55945-73-8		*Chaetomium globosum*		[49]
46	Chaetoglobosin E	55945-74-9		*Chaetomium globosum* (CDW7)		[49]
47	Chaetoglobosin F	55945-75-0		*Chaetomium globosum* (CDW7)		[47]
48	Chaetoglobosin Fa	1599426-06-8		*Chaetomium globosum*		[47]
49	Chaetoglobosin Fex	149457-95-4		*Chaetomium globosum*		[47]
50	Chaetoglobosin G	65773-98-0		*Chaetomium globosum* (NM0066)		[47]
51	Chaetoglobosin R	777939-30-7		*Chaetomium globosum*		[49]
52	Chaetoglobosin V	1399682-37-1		*Chaetomium globosum*		[47]
53	Chaetoglobosin Vb	1399690-75-5		*Chaetomium globosum* (CDW7)		[48]
54	Chaetoglobosin Y	1608108-89-9		*Chaetomium globosum*		[48]
55	Chaetomugilide A	1418138-71-2		*Chaetomium globosum*		[45, 47]
56	Chaetomugilide B	1433976-48-7		*Chaetomium globosum*		[45]
57	Chaetomugilide C	1418138-70-1		*Chaetomium globosum*		[45, 47]
58	Chaetomugilin A	1041640-66-7		*Chaetomium globosum*		[45]
59	Chaetomugilin D	1098081-38-9		*Chaetomium globosum*		[25]
60	Chaetomugilin I	1187848-00-5		*Chaetomium globosum*		[25]
61	Chaetomugilin J	1187848-01-6		*Chaetomium globosum*		[25]
62	Chaetomugilin O	1187848-06-1		*Chaetomium globosum*		[25]
63	Chaetomugilin Q	1319729-85-5		*Chaetomium globosum*		[25]
64	Chaetomugilin S	1399093-77-6		*Chaetomium globosum*		[25]
65	Chaetoviridin C	128230-02-4		*Chaetomium globosum*		[15]
66	Chaetoviridin D	128230-04-6		*Chaetomium globosum*		[33]
67	Chaetoviridin E	1178875-15-4		*Chaetomium globosum*		[33]
68	Cyclo-(Phe-Gly)	5037-75-2		*Chaetomium globosum*		[33]
69	Cyclopentadecane	295-48-7		*Chaetomium globosum* No.16		[45]
70	Dimethyl phthalate	131-11-3		*Chaetomium globosum* No.16	Used in plastics, insect repellents, safety glass, and lacquer coatings	[45]
71	Epimwsokorwnone A	1073-96-7		*Chaetomium globosum*		[33]
72	Ergosta-4 6,8,22-tetraen-3-one/ergosta-4,6,8,22-tetraen-3-one	194721-75-0		*Chaetomium globosum* (ZY-22)		[33]
73	Ergosterol	57-87-4		*Chaetomium globosum*	Formation of vitamin D2	[49]
74	Ergosterol peroxide (5α,8α-epi-dioxy-(22E,24R) -ergosta-6,22-dien-3β-ol)	2061-64-5		*Chaetomium globosum*	An antineoplastic agent, an antimycobacterial drug and a trypanocidal drug	[33]
75	Ethanoic acid	64-19-7		*Chaetomium globosum* T16	Food additive, and in petroleum production	[46]
76	Ethyl 13-methyl-tetradecanoate	64317-63-1		*Chaetomium globosum* No. 16		[45]
77	Ethyl 2-heptenoate	2351-88-4		*Chaetomium globosum* T16		[45]
78	Ethylidene acetate	542-10-9		*Chaetomium globosum* T16		[45]
79	flavipin (1,2-benzenedicarboxaldehyde-3,4,5-trihydroxy-6-methyl)	483-53-4		*Chaetomium globosum* CDW7	Antioxidant fungicides	[22]
80	Fumigaclavine B	6879-93-2		*Chaetomium globosum*		[47]
81	Fumitremorgin C	118974-02-0		*Chaetomium globosum* (NM0066)	A mycotoxin and a breast cancer resistance protein inhibitor	[33]
82	Gliotoxin	67-99-2		*Chaetomium globosum* (NM0066)	A mycotoxin, an immunosuppressive agent, an protein farnesyltransferase inhibitor, a proteasome inhibitor and an antifungal agent	[33]
83	Globosterol	1193319-70-8		*Chaetomium globosum* ZY-22		[44]
84	Glycerol formal	5464-28-8		*Chaetomium globosum* T16		[46]
85	Hexadecane	544-76-3		*Chaetomium globosum*	Used as a solvent and an ingredient in gasoline and diesel and jet fuels	[45]
86	Hexadecanoic acid, ethyl ester	628-97-7		*Chaetomium globosum* No. 16	Used as softener, lubricant, food additive	[45]
87	Hexadecanoic acid, methyl ester	112-39-0		*Chaetomium globosum* No. 16	Used as intermediate of emulsifier, wetting agent, stabilizer and plasticizer	[45]
88	Indole-3- carboxylic acid	771-50-6		*Chaetomium globosum* ZY-22	Used for synthesis of to rise tron and antiviral drugs	[33]
89	Indole-3-acetic acid	87-51-4		*Chaetomium globosum*	Plant growth stimulating hormone	[33]
90	Isopentyl alcohol, acetate	123-92-2		*Chaetomium globosum* T16	Used as a solvent and preparation of a variety of flavor food flavor	[22]
91	Lactic acid	50-21-5		*Chaetomium globosum* T16	Used to make some plasticizers, adhesives, pharmaceuticals and salts, used in the leather tanning industry and as a solvent	[46]
92	Lactic acid, 2-methyl-,ethyl ester	80-55-7		*Chaetomium globosum* T16		[46]
93	Maltol	118-71-8		*Chaetomium globosum* MX-0510	Food additive	[33]
94	Mannitol	87-78-5		*Chaetomium globosum*	Used as an osmotic diuretic	[33]
95	Methyl 13-methyltetradecanoate	5129-59-9		*Chaetomium globosum* No. 16		[45]
96	Methyl 9,12-heptadecadienoate	15620-59-4		*Chaetomium globosum* No. 16		[45]
97	Methyl vinylcarbinol	598-32-3		*Chaetomium globosum*	Food additive	[46]
98	Methylthiogliotoxin	74149-38-5		*Chaetomium globosum* (NM0066)		[33]
99	*o*-Coumaric acid	583-17-5		*Chaetomium globosum* ZY-22	An antioxidant and is believed to reduce the risk of stomach cancer by reducing the formation of carcinogenic nitrosamines	[33]
100	Octanoic acid, methyl ester	111-11-5		*Chaetomium globosum* No. 16	Food additive	[45]
101	Pentadecane	629-62-9		*Chaetomium globosum* No. 16	Used as a solvent and in some household pesticides	[45]
102	Pentadecanoic acid, methyl ester	7132-64-1		*Chaetomium globosum* No. 16	Fuels and fuel additives Intermediates, pesticide	[45]
103	*p*-Hydroxybenzoic acid	99-96-7		*Chaetomium globosum*	Used as preservatives, fungicides	[33]
104	Pseurotin A	58523-30-1		*Chaetomium globosum* (NM0066)	An azaspiro compound, an oxaspiro compound and a lactam	[33]
105	Quercetin	117-39-5		*Chaetomium globosum* GCZX015	Combined with chemotherapeutic drugs, produces anti-inflammatory and anti-allergy effects	[33]
106	Squalene	111-02-4		*Chaetomium globosum* (NM0066)	Investigated as an adjunctive cancer therapy, also used as cosmetics and dietary supplement	[33]
107	*S*-Tetrachloroethane	79-34-5		*Chaetomium globosum* T16	Used to make paint, varnish and rust removers, as a solvent and as an ingredient in pesticides	[45]
108	Succinic acid	110-15-6		*Chaetomium globosum*	A radiation protective agent, an anti-ulcer drug	[33]
109	Tetradecane	629-59-4		*Chaetomium globosum* No.16	Used as a solvent and some pesticide sprays	[45]
110	Thymine	65-71-4		*Chaetomium globosum* ZY-22	A pyrimidine nucleobase and a pyrimidone	[33]
111	Tridecane	629-50-5		*Chaetomium globosum* No. 16	Used as a solvent and as an ingredient in gasoline and diesel and jet fuel	[45]
112	Triethylene glycol monomethyl ether acetate	3610-27-3		*Chaetomium globosum* T16		[46]
113	Uracil	66-22-8		*Chaetomium globosum* ZY-22	Use in the body to help synthesis of many enzymes, and the biosynthesis of polysaccharides and the transportation of sugars containing aldehydes	[49]
114	α-Guajene	3691-12-1		*Chaetomium globosum* No. 16		[45]

Chaetomugilin A and D, both are a kind of azaphilone isolated from Chaetomium globosum and has been shown to exhibit inhibitory activity against the brine shrimp (Artemia salina) and Mucor miehei [22]. Chaetomugilide A isolated from *Chaetomium globosum* TY1 has strong activity against hepatoma cell HepG-2, and the IC_50_ value is only 1.7 μmol/L [23]. Chaetoglobosin A is a *Chaetomium* secretion with the anticancer activity in vitro [24] and it derivates into other bilobalide compounds MBJ-0038, MBJ-0039, and MBJ-0040 [25]. Chaetoglobosin E is a cytochalasan alkaloid found in *Chaetomium globosum* and *Chaetomium subaffine*. It is a cytochalasan alkaloid, a member of indoles, a macrocycle and a secondary alpha-hydroxy ketone. It has a role as a *Chaetomium* metabolite and an antineoplastic agent.

One new cytochalasan alkaloid, chaetoglobosin V(b), together with two structurally related known compounds, chaetoglobosin V and chaetoglobosin G, were isolated from the ethyl acetate extract of a culture of the endophytic fungus *Chaetomium globosum*, associated with the leaves of *G. biloba* tree. The structures of the isolated compounds were elucidated by spectroscopic methods including 1D and 2D NMR and mass spectrometry. The absolute conStruration of chaetoglobosin V(b) was established by means of electronic circular dichroism (CD) spectroscopy. The correlation between compounds was demonstrated by a biomimetic transformation of chaetoglobosin G under mild conditions in chaetoglobosins V and V(b). The isolated metabolites were tested against some phytopathogens [22].

The compound flavipin isolated from *Chaetomium globosum* CDW 7 has strong antioxidant activity [23]. *Chaetomium globosum* ZY-22 could produce two polyhydroxylated steroids [24] and two other important compounds bilobalide, ginkgolides are to be beneficial to human health [26]. Bilobalide has neuroprotective effects [27] as well as inducing the liver enzymes CYP3A1 and 1A2 which may be partially responsible for interactions between gingko and other herbal medicines or pharmaceutical drugs; while ginkgolide has been investigated for its potential to reducing migraine frequency [28]. Ergosterol peroxide (5α,8α-epidioxy-22E-ergosta-6,22-dien-3β-ol) is a steroid derivative. It has been reported to exhibit immune- suppressive, anti-inflammatory, antiviral, trypanocidal and antitumor activities in vitro [27].

### Secondary metabolites of *Aspergillus*

*Aspergillus* is the dominant flora of endophytic fungi of *G. biloba* and was isolated from different parts of *G. biloba* which cultivated in various areas. A total of 44 metabolites were found in the fermentation broth of *Aspergillus* (see Table 2), among which 3-hydroxy-terphenyl, 4,5-dimethoxycandidusin A, prenylcandidusin C, and prenylterphenyllin were studied most popularly. For 4″-Deoxycandidusin A, 4″-deoxytripentin, 4′-deoxy-3-hydroxyrisperidone, aspergiloid A, coumarin A, and tribenzine, three articles reported about each compound, respectively. Among these metabolites, 3-hydroxy-terphenyl and 4″-deoxycandidusin A, 4″-deoxytripentin have strong inhibitory activity against neuraminidase [29]; 4′-deoxy-3-hydroxytripentin, 3-hydroxy-terphenyl, 4″-deoxycandidusin has moderate activity against human nasopharyngeal carcinoma cell KB, human gastric cancer cell SGC-7901, human colon cancer cell SW1116 and human lung cancer cell A549 [30].

**Table 2:** Secondary metabolites of *Aspergilus* in *Ginkgo biloba*

No.	Metabolites	CAS number	Molecular structure	Endophytes	Application	References
1	3-Hydroxyterphenyllin	66163-76-6		*Aspergillus* sp.	Induces apoptosis and S phase arrest in human ovarian carcinoma cells	[28, 50]
2	4″-Deoxycandidusin A	1354549-88-4		*Aspergillus* sp.		[51, 52]
3	4″-Deoxyterphenyllin	59904-04-0		*Aspergillus* sp.		[50]
4	4,5-Dimethoxycandidusin A/3,4-dimethoxycandidusin A	1354549-89-5		*Aspergillus* sp.		[50, 52]
5	4′-Deoxy- 3-hydroxyterphenyllin	1296205-84-9		*Aspergillus* sp.		[50, 52]
6	4′’-Deoxy-5′-desmethyl-terphenyllin	1354549-87-3		*Aspergillus* sp.		[50]
7	4′’-Deoxyprenylterphenyllin	959124-87-9		*Aspergillus* sp. IFB-YXS	Potential anticancer lead molecules	[50]
8	4-Hydroxy-3-(3′-methyl-2′-butenyl) benzoic acid	1138-41-6		*Aspergillus* sp. YXf3	Show potent inhibition of HLE	[50]
9	5′-Desmethylterphenyllin	1299485-87-2		*Aspergillus* sp.	An alpha-glucosidase inhibitor	[50]
10	Alternariol	641-38-3		*Aspergillus* sp. YXf3	An cholinesterase inhibitor and a mycotoxin	[52]
11	Alternariol monomethyl ether/alternariol-4-methyl ether	23452-05-3		*Aspergillus* sp. YXf3	An antifungal agent	[52]
12	Aspergiloid A	1354549-91-9		*Aspergillus* sp.		[50]
13	Aspergiloid B	1354549-92-0		*Aspergillus* sp.		[50]
14	Aspergiloid C	1354549-93-1		*Aspergillus* sp.		[50]
15	Aspergiloid D	1354549-94-2		*Aspergillus* sp.		[50]
16	Aspergiloid E	1579256-33-9		*Aspergillus* sp. YXf3		[52]
17	Aspergiloid F	1579256-35-1		*Aspergillus* sp. YXf3		[52]
18	Aspergiloid G	1579256-37-3		*Aspergillus* sp. YXf3		[52]
19	Aspergiloid H	1579256-39-5		*Aspergillus* sp. YXf3		[52]
20	Aspergiloid I	1887750-59-5		*Aspergillus* sp. YXf3	Anti-cancer and inhibition of plant pathogens	[50]
21	Candidusin A	81474-59-1		*Aspergillus* sp.		[50]
22	Candidusin C/4″-methoxycandidusin A	267007-58-9		*Aspergillus* sp.		[50]
23	Chlorflavonin	23363-64-6		*Aspergillus* sp. (strain no. YXf3)	An antifungal agent	[50]
24	Chlorflavonin A	1443055-96-6		*Aspergillus* sp. (strain no. YXf3)	An antifungal agent	[50]
25	Cyclo-(L-Leu-L-Trp)	15136-34-2		*Aspergillus* sp. YXf3		[50]
26	Ginkgolide B	15291-77-7		*Aspergillus.fumigatus var. fumigatus* FG 05	Ginkgolide B protects human umbilical vein endothelial cells against xenobiotic injuries via PXR activation	[52]
27	Ginkgolide C	15291-76-6		*Aspergillus*		[32]
28	Prenylcandidusin B	1297472-19-5		*Aspergillus* sp. IFB-YXS	An antineoplastic agent	[53]
29	Prenylcandidusin C	1297472-20-8		*Aspergillus* sp.	An antineoplastic agent	[53]
30	Prenylterphenyllin	959124-85-7		*Aspergillus* sp.	Exhibits cytotoxic activity, an antineoplastic agent	[53]
31	Prenylterphenyllin B	1297472-16-2		*Aspergillus* sp. IFB-YXS	Exhibits cytotoxic activity, an antineoplastic agent	[53]
32	Sphaeropsidin A	38991-80-9		*Aspergillus* sp. YXf3	larvicidal and biting deterrents against *Aedes aegypti*	[50]
33	Sphaeropsidin B	39022-38-3		*Aspergillus* sp. YXf3		[50]
34	Terphenolide	1354549-90-8		*Aspergillus* sp.		[50]
35	Terphenyllin	52452-60-5		*Aspergillus* sp.	A mycotoxin	[50]
36	Terreinol	669073-67-0		*Aspergillus* sp. YXf3		[31]
37	Xanthoascin	61391-08-0		*Aspergillus* sp. IFB-YXS		[53]
38	Prenylterphenyllin D	2079979-59-0		*Aspergillus* sp. IFB-YXS	Antibacterial activities, anti-phytopathogenic activities	[31]
39	Prenylterphenyllin E	2079979-60-3		*Aspergillus* sp. IFB-YXS	Antibacterial activities, anti-phytopathogenic activities	[31]
40	2′-O-Methylprenylterphenyllin	2079979-61-4		*Aspergillus* sp. IFB-YXS	Antibacterial activities, anti-phytopathogenic activities	[31]
41	4-O-Methylprenylterphenyllin	2079979-62-5		*Aspergillus* sp. IFB-YXS		[31]
42	[1,1′:4′,1′’-Terphenyl]-4,4′’-diol, 2′,3′,5′-trimethoxy-(9CI)	59914-89-5		*Aspergillus* sp. IFB-YXS		[31]
43	[1,1′:4′,1′’-Terphenyl]-2′,4′’-diol,3′,4,6′-trimethoxy-(9CI)	59903-93-4		*Aspergillus* sp. IFB-YXS		[31]
44	[1,1′:4′,1′’-Terphenyl]-2′,4-diol,3′,4′’,6′-trimethoxy-(9CI)	59903-92-3		*Aspergillus* sp. IFB-YXS		[31]

### Secondary metabolites of *Alternaria*

*Alternaria* is a very common fungus. It is an important pathogen for plants, human and animal diseases. It is a biological resource with great application potential as well. According to the existing literatures, 17 metabolites were isolated from the fermentation products of *Alternaria* (see Table 3). Alterperylenol inhibits human telomerase activity. Alterperylenol can inhibit telomerase activity (IC_50_ = 30 μM), but altertoxin I (dihydroalterperylenol), a structurally related compound, did not affect activity at 1 mM. Moreover, alterperylenol and altertoxin I show phytotoxic and antifungal activity [31].

**Table 3:** Secondary metabolites of *Alternaria* in *Ginkgo biloba*

No.	Metabolites	CAS number	Molecular structure	Endophytes	Application	References
1	(22E,24R)-ergosta-7,22-diene-3β,5α,6β-triol/cerevisterol	516-37-0		*Alternaria tenuissima* SY-P-07		[29]
2	(2R,3R)-3,5,7,3′,5′-pentahydroxyflavane	87592-94-7		*Alternaria tenuissima* SY-P-07		[29]
3	3β,5α,9α-Trihydroxy-(22E,24R)-ergosta-7,22-dien-6-one	88191-14-4		*Alternaria tenuissima* SY-P-07		[29]
4	6-Epi-stemphytriol	1262797-65-8		*Alternaria tenuissima* SY-P-07		[29]
5	7-Epi-8-hydroxyaltertoxin I	1262797-64-7		*Alternaria tenuissima* SY-P-07		[29]
6	Alternariol	641-38-3		*Alternaria* No. 28	An cholinesterase inhibitor	[29]
7	Alternariol monomethyl ether/alternariol-4-methyl ether	23452-05-3		*Alternaria* No. 28	An antifungal agent	[29]
8	Alterperylenol	88899-62-1		*Alternaria tenuissima*		[45]
9	Altertoxin I (dihydroalterperylenol)	56258-32-3		*Alternaria* sp.		[29]
10	Ergosta-4,6,8,22-tetraen-3-one/ergosta-4,6,8,22-tetraen-3-one	194721-75-0		*Alternaria* No. 28		[29]
11	Ergosterol	57-87-4		*Alternaria* sp.	Formation of vitamin D2	[29]
12	Flazin	100041-05-2		*Alternaria tenuissima* SY-P-07		[47]
13	Solanapyrone G	220924-51-6		*Alternaria tenuissima* SY-P-07		[47]
14	Stemphyperylenol	102694-33-7		*Alternaria tenuissima* SY-P-07	An antifungal agent	[47]
15	Tenuazonic acid	610-88-8		*Alternaria* No. 28	An antibiotic with antiviral and antineoplastic, also as a mycotoxin	[29]
16	Vivotoxin II	1261267-71-3		*Alternaria* No. 28		[29]

In these metabolites, botulinum toxin and botulinum toxin II have strong cytotoxic activity. When the concentration is 10 μg/mL, the mortality rate of brine shrimp is 68.9% and 73.6%, respectively [32]. *Alternaria* No. 28 could produce cytotoxic metabolites which have inhibitory potential against some different protein kinases [7].

### Secondary metabolites of *Penicillium*

*Penicillium* is widely distributed in nature and generally has a strong biological activity. According to the existing literatures, 17 secondary metabolites were found from the fermentation products of *Penicillium* sp. in *G. biloba* (Table 4), and some metabolites were biologically active. The compound arcacic acid is isolated from the fermentation broth of *Penicillium commune*, which has antibacterial activity and has inhibition activities on 12 kinds of plant pathogens, especially has strong inhibitory activity against *Bacillus licheniformis* and *Sclerotinia sclerotiorum*, and the IC_50_ values are only 39.28 mg/L and 60.62 mg/L [33].

**Table 4:** Secondary metabolite of *Penicillium* in *Ginkgo biloba*

No.	Metabolites	CAS number	Molecular structure	Endophytes	Application	References
1	2′-Deoxyuridine/uracil deoxyriboside	951-78-0		*Penicillium* sp. YY-25	Antimetabolite	[29]
2	3-Methylorsellinic acid	4707-46-4		*Penicillium* No. 97	Antibacterial activity	[29]
3	3-Methylpiperazine-2,5-dione	6062-46-0		*Penicillium* sp. YY-24		[29]
4	Adenine	73-24-5		*Penicillium* sp. YY-22	Dietary supplement	[29]
5	Adenosine	58-61-7		*Penicillium* sp. YY-20	Analgesic, antiarrhythmic	[29]
6	Anthranilamide	88-68-6		*Penicillium* No. 97	Fluorescent dyes	[54]
7	Anthranilic acid	118-92-3		*Penicillium* No. 97	Anticonvulsants	[55]
8	Cyclopaldic acid	477-99-6		*Penicillium commune* (TMSF169)		[56]
9	Ferulic acid	1135-24-6		*Penicillium* No. 97	Free radical scavengers, anti-inflammatory agents, antihypertensive agents, anticoagulants	[55]
10	Fructigenine A	144606-96-2		*Penicillium* No. 97	Inhibits the growth of leukemia cells	[55]
11	Indole-3-acetic acid	87-51-4		*Penicillium* No. 97	Used for preventing, destroying or mitigating pests	[55]
12	Methyl β-d-ribofuranoside	7473-45-2		*Penicillium* sp. YY-21	Used to synthesize novel alpha-amino acid esters against herpes simplex virus 1 (hsv-1) and hepatitis b virus	[29]
13	Orsellinic acid	480-64-8		*Penicillium* No. 97		[29]
14	*p*-Hydroxybenzoic acid	99-96-7		*Penicillium* No. 97		[55]
15	β-sitosterol	83-46-5		*Penicillium* No. 97	Hypolipidemic agents	[55]
16	Quercetin glycoside (orange pigment)	3520-72-7		*Penicillium* sp.		[34]

The compounds adenosine, deoxyadenosine and adenine which were isolated from the fermentation product of *Penicillium* sp. YY-20 have a strong scavenging capacity for DPPH free radical [34]. Wu isolated *Penicillium cataractum* SYPF 7131 from 58 endophytic fungi obtained from the leaves, stems and roots of *G. biloba*. This strain displayed the strongest antibacterial activity [35].

### Secondary metabolites of *Xylaria*

43 kinds of compounds were isolated from the fermentation products of *Xylaria* in *Ginkgo biloba* (Table 5), in which the compound 7-amino-4-methylcoumarin was isolated from the fermentation product of Xylaria sp. YX-28 [36]. It has antibacterial activity and also has strong inhibitory activity against 13 kinds of human susceptible pathogens, which is significantly higher than the positive controls ampicillin, gentamicin and tetracycline.

**Table 5:** Secondary metabolite of *Xylaria* in *Ginkgo biloba*

No.	Metabolites	CAS number	Molecular structure	Endophytes	Application	References
1	7-Amino-4-methylcoumarin	26093-31-2		*Xylaria* sp. YX-28	A fluorescent dye used to stain biological specimens	[57]
2	Pentadecane	629-62-9		*Xylaria* sp. YX-28	Treatment of plantar keratosis with medicinal plant in diabetic patients	[57]
3	Quercetin	117-39-5		*Xylaria Colletotrichum*	Chemotherapy induced oral mucositis; treatment of erosive and atrophic oral lichen planus; chronic obstructive pulmonary disease; gastroesophageal reflux disease	[57]
4	Tetradecane	629-59-4		*Xylaria* sp.YX-28		[57]
5	Tridecane	629-50-5		*Xylaria* sp. YX-28		[57]
6	Dibutyl phthalate	84-74-2		*Xylaria* sp. YX-28	Against the larval trombiculid mite; preventing scrub typhus of topical application in troops	[57]
7	1,3-Diphenyl-2-pyrazoline	2538-52-5		*Xylaria* sp. YX-28		[57]
8	1-Acetyl-1,2,3,4-tetrahydropyridine	19615-27-1		*Xylaria* sp. YX-28		[57]
9	Z,Z-7,11-Hexadecadien-1-ol	53963-06-7		*Xylaria* sp. YX-28		[57]
10	Isosorbide	652-67-5		*Xylaria* sp. YX-28	Prevention of angina pectoris due to coronary artery disease; short-term reduction of intraocular pressure	[57]
11	Dimethoxy-phenol	91-10-1		*Xylaria* sp.YX-28	Food Flavoring Agents	[57]
12	1-hydroxymethyl-1,2,3,4,-tetrahydro-naphthalen-2-ol	872824-43-6		*Xylaria* sp. YX-28		[57]
13	(1,4-Dimethylpent-2-enyl)benzene	951288-80-5		*Xylaria* sp. YX-28		[57]
14	2,4-Bis(1,1-dimethylethyl)phenol	96-76-4		*Xylaria* sp. YX-28		[57]
15	3-Phenyl-4-methyl-isoxazol-5(4)-one	875244-90-9		*Xylaria* sp. YX-28		[57]
16	3,4-Dihydro-8-hydroxy-3-methyl-isocoumarin	1200-93-7		*Xylaria* sp. YX-28		[57]
17	[l(3-butenylthio)-2-nitroethyl]-benzene	128869-50-1		*Xylaria* sp. YX-28		[57]
18	Pentadecanoic acid, methyl ester	7132-64-1		*Xylaria* sp. YX-28	pesticide	[57]
19	14-Octadecenal	56554-89-3		*Xylaria* sp. YX-28		[57]
20	E-11,13-Dimethyl-12-tetradecen-1-ol acetate	400037-00-5		*Xylaria* sp. YX-28		[57]
21	Hexadecanoic acid, methyl ester	112-39-0		*Xylaria* sp. YX-28	Food flavoring agents	[57]
22	*n*-Hexadecanoic acid	57-10-3		*Xylaria* sp. YX-28	Inhibits HIV-1 infection; a potential candidate for specifically attack multiple myeloma cells	[57]
23	2-Undecenal	2463-77-6		*Xylaria* sp. YX-28		[57]
24	Hexadecanoic acid, 14-methyl-methyl ester	2490-49-5		*Xylaria* sp. YX-28		[57]
25	9,12-Octadecadienoic acid(Z,Z)-methyl ester	112-63-0		*Xylaria* sp. YX-28	Flavoring agent or adjuvant	[57]
26	9-Octadecenoic acid (Z)-,methyl ester	112-62-9		*Xylaria* sp. YX-28	Solvents	[57]
27	3,7,11-trimethyl-2,6,10-Dodecatrien-1-ol	4602-84-0		*Xylaria* sp. YX-28	Inhibits proliferation and induces apoptosis of tumour-derived but not non-transformed cell lines	[57]
28	9,12-Octadecadienoic acid (Z,Z)	2197-37-7		*Xylaria* sp. YX-28	Treats the prevention of preeclampsia;	[57]
29	9-Octadecenamide (Z)	3322-62-1		*Xylaria* sp. YX-28	Induce drowsiness or sleep or to reduce psychological excitement or anxiety	[57]
30	Pentadecanoic acid,2-hydroxymethy l ester	98863-01-5		*Xylaria* sp. YX-28	Emulsifier	[57]
31	Ferruginol	514-62-5		*Xylaria* sp. YX-28	An antineoplastic agent; antibacterial agent; protective agent	[57]
32	9,12-Octadecadienoic acid(Z,Z)-,2-hydroxy-1-(hydroxy methyl)ethyl ester	544-35-4		*Xylaria* sp. YX-28	Flavoring agents	[57]
33	Hexadecanoic acid, 2-hydroxy-1-(hydroxymethyl)ethyl ester	23470-00-0		*Xylaria* sp. YX-28	Lipid maps classification	[57]
34	Bis(2-ethylhexyl)phthalate	117-81-7		*Xylaria* sp.YX-28		[57]
35	5,6,8,9,10,11-Hexahydrobenz[A]anthracene	67064-61-3		*Xylaria* sp. YX-28		[57]
36	1,2,3,4-Tetrahydro-Triphenylene	5981-10-2		*Xylaria* sp. YX-28		[57]

### Secondary metabolites of *Fusarium*

*Fusarium* is one of the dominant bacteria, which can be isolated from different parts of *Ginkgo* cultivated in various areas. According to the literatures, 25 kinds of compounds were isolated from the fermentation products of *Fusarium* (Table 6). Since *Fusarium* of *G. biloba* can produce ginkgolides B, it can be used as a new source of ginkgolides B [37]. Some studies have shown that *Fusarium oxysporum* GF521 can produce rutin and kaempferol, and the total flavonoids production of endophytic fungi is 21.10 ± 1.30 mg/L, which indicates that *Fusarium* genus also have a high ability of producing flavonoids [37].

**Table 6:** Secondary metabolite of *Fusarium* in *Ginkgo biloba*

No.	Metabolites	CAS number	Molecular structure	Endophytes	Application	References
1	Adenosine	58-61-7		*Fusarium solani* GBT07 GBT07	Terminate paroxysmal supraventricular tachycardia; terminating stable and narrow-complex supraventricular tachycardias; adjunct to thallous chloride TI 201 myocardial perfusion scintigraphy and vagal maneuvers and clinical assessment	[11]
2	Benzeneethanol/Phenylethyl alcohol	60-12-8		*Fusarium* sp. G1024	Anti-infective agents, local; disinfectants; preservatives, pharmaceutical	[11]
3	Enniatin B	917-13-5		*Fusarium* sp.		[58]
4	Ginkgolide B	15291-77-7		*Fusarium oxysporum*		[59, 60]
5	Hexadecane	544-76-3		*Fusarium* sp. G1024		[11]
6	Kaempferide	491-54-3		*Fusarium solani*	An antihypertensive agent	[61]
7	Kaempferol	520-18-3		*Fusarium oxysporum*	A possible cancer treatment; antibacterial agent	[61]
8	Quercetin	117-39-5		*Fusarium oxysporum*		[57]
9	Rutin	153-18-4		*Fusarium oxysporum*	A role as an antioxidant; antiallergic; anti-inflammatory; antiproliferative; and anticarcinogenic properties	[61]
10	Soyasapogenol B	595-15-3		*Fusarium oxysporum Schlecht* GB-1(3)		[61]
11	Tetradecane	629-59-4		*Fusarium* sp. G1024		[11]
12	β-Sitosterol	83-46-5		*Fusarium oxysporum Schlecht* GB-1(3)	As anticholesteremic drug; antioxidant; treats hyperlipidemia.	[61]
13	Isorhamnetin	480-19-3		*Fusarium* sp	Warning; (tyrosinase inhibitor; an anticoagulant)	[62]
14	Decane	124-18-5		*Fusarium* sp. G1024		[11]
15	2-Ethyl-1-hexanol	104-76-7		*Fusarium* sp. G1024		[11]
16	2-Butanol,3,3′-oxybis-4-ethylphenol	123-07-9		*Fusarium* sp. G1024	Flavoring Agents	[11]
17	Dodecane	112-40-3		*Fusarium* sp. G1024	Increase the risk of neoplasms in humans or animals	[11]
18	1,2-benzisothiazole	272-16-2		*Fusarium* sp. G1024		[11]
19	4-Ethyl-2-methoxyphenol	2785-89-9		*Fusarium* sp. G1024	Flavoring agents	[11]
20	*p*-Nitroacetophenone	100-19-6		*Fusarium* sp. G1024	Potentiate the effectiveness of radiation therapy in destroying unwanted cells	[11]
21	2,3,5,6-Tetramethyl-p-benzoquinone	527-17-3		*Fusarium* sp. G1024	product quinones duroquinone	[11]
22	Eicosane	112-95-8		*Fusarium* sp. G1024	Flavoring Agents.	[11]
23	1,2-Benzenedicarboxylic acid bis(2-methylpropyl)ester	88-99-3		*Fusarium* sp. G1024		[11]
24	Dibutyl phthalate	84-74-2		*Fusarium* sp. G1024	Against the larval trombiculid mite; preventing scrub typhus of topical application in troops	[11]

### Secondary metabolites of other genus

53 compounds were isolated from the fermentation products of other genus in *G. biloba* (Table 7), some of which can also produce other valuable compounds. From the endophytic *Muscodor albus* GBA, 19 kinds of volatile components can be separated [24], which normally have a strong ecological effect. Some volatile components can inhibit the pathogenic microorganisms and enhance the disease resistance of plants. *Bacillus amyloliquefaciens* can produce 8 kinds of compounds [35, 37] which have some biological activities. Two compounds, apigenin-8-C-glucoside and 2-(Hydroxymethylthio) ethanol, were isolated from *Colletotrichum* sp. NTB-2., in which apigenin-8-C-glucoside has strong inhibitory activity against *Bacillus subtilis*, *Salmonella typhimurium* and *Pseudomonas cepacia* [38]. Moreover, *Colletotrichum* sp. could produce flavones which exhibited potent anti-cancer, anti-HIV [39] and antioxidant activities [40].

**Table 7:** Secondary metabolite of other endophytics in *Ginkgo biloba*

No.	Metabolites	CAS number	Molecular structure	Endophytes	Application	References
1	2-(Hydroxymethylthio)ethanol	876503-58-1		*Colletotrichum* sp. NTB-2	Platelet aggregation inhibitor, an alpha-glucosidase inhibitor, an antineoplastic agent	[63]
2	Apigenin-8-C-β-d-glucopyranoside	3681-93-4		*Colletotrichum* sp.		[63, 64]
3	6-Ethoxyl-2,4-amide lactone			*Bacillus amyloliquefaciens* CGMCC 5569		[64]
4	6-Hydroxylbutyl-2,4-amide lactone			*Bacillus amyloliquefaciens* CGMCC 5569		[64]
5	6-Hydroxypropyl-2,4-amide lactone			*Bacillus amyloliquefaciens* CGMCC 5569		[64]
6	Biuret	108-19-0		*Bacillus amyloliquefaciens* CGMCC 5569	Used for preventing, destroying or mitigating pests	[64]
7	Ginkgolide B	15291-77-7		*Oospora wallr.* G10	Fibrinolytic agents	[65]
8	2′-Deoxyuridine/uracil deoxyriboside	951-78-0		Unidentified	Antimetabolites	[65]
9	3-Methylpiperazine-2,5-dione	6062-46-0		Unidentified		[65]
10	Adenine	73-24-5		Unidentified		[65]
11	Adenine deoxyriboside			Unidentified		[65]
12	Adenosine	58-61-7		Unidentified	Used as an initial treatment for the termination of paroxysmal Supraventricular tachycardia	[65]
13	Quercetin	117-39-5		*Stemphylium* sp. Act inomyces	Antioxidants	[37, 66]
				*Nodulisporium hyalosporum* *Schizophyllum commune* Fr.		[67]
				*Fusella Sacc* *Alternaria* sp *Sphacelia* sp. *Anpelomyces humuli*		[37]
				*Phoma glomerate*		[30, 61]
				*Trichothecium*		[53]
				*Mucor circinelloides*		[40]
				*Sphaeropsis* sp. B301		[68]
14	Kaempferol	520-18-3		*Fusella Sacc* *Alternaria* sp. *Gibberella* sp. *Sphacelia* sp. *Dematium Pers*	As a selective estrogen receptor modulator	[66]
				*Trichothecium*		[53]
				*Sphaeropsis* sp.		[68]
15	Cerebroside B	88642-46-0		*Phyllosticta* sp. TP78, (GenBank ID: KC445736)	An antimicrobial compound	[20, 21]
16	Cerebroside C	98677-33-9		*Phyllosticta* sp. TP78 (GenBank ID: KC445736)	Increases tolerance to chilling injury and alters lipid composition in wheat roots	[20, 21]
17	Enniatin B1	19914-20-6		*Tuberculariaceae* F1-3	Fusarium mycotoxins	[69]
18	Enniatin D	19893-21-1		*Tuberculariaceae* sp. F1-3	Inhibition of Botrytis cinerea spore germination	[69]
19	Benzeneethanol/Phenylethyl alcohol	60-12-8		*Muscodor albus* strain GBA	Anti-bacterial agents and antioxidants. Anti-Infective Agents	[69]
20	Ginkgolide C	15291-76-6		*Gloeosporium; Tolura; Phacodium*	Reduced lipid accumulation and suppresses adipogenesis	[32]
21	Kaempferide	491-54-3		*Phoma glomerata*	Reverse bacterial resistance to amoxicillin in AREC	[61]
				*Anpelomyces humuli*		[61]
22	Rutin	153-18-4		*Mucor circinelloides* GF521	Used therapeutically to decrease capillary fragility	[61]
				*Nodulisporium hyalosporum*		[67]
23	Sporothriolide	154799-92-5		*Nodulisporium* sp. A21	Used to treat the infection caused by candida albicans and cryptococcus neoformans	[55]
24	Isorhamnetin	480-19-3		*Stemphylium* sp. *Alternaria* sp *Gibberella* sp. *Trichothecium*	prevents endothelial dysfunction, superoxide production, Isorhamnetin appears to be a potent drug against esophageal cancer	[62]
				*sphaeropsis*		[68]
				*Plantactinospora* sp. NEAU-gxj3		[20, 21]
25	Antibiotic U-62162	82516-67-4		*Plantactinospora* sp. NEAU-gxj3	Inhibited the growth of Gram-positive bacteria	[20, 21]
26	Salternamide C	1662688-81-4		*sphaeropsis*		[68]
27	Abscisic acid	21293-29-8		*Phoma betae*	Plant Growth Regulator	[69]
28	Taxol	33069-62-4		*Phomopsis* sp. 2 strain BKH 30 (BSL No. 72)	An antineoplastic agent, tubulin modulators	[70]
				*Muscodor albus* strain GBA		[69]
29	Acetic acid, methyl ester	79-20-9		*Muscodor albus* strain GBA		[69]
30	2-Butanone	78-93-3		*Muscodor albus* strain GBA	Polar aprotic solvent	[69]
31	Acetic acid, 2-methylpropyl ester	110-19-0		*Muscodor albus* strain GBA	An antifungal agent	[71]
32	1-Propanol, 2-methyl	78-83-1		*Muscodor albus* strain GBA	Possesses nicotine-like synaptotropic actions on the nervous systems	[71]
33	1-Butanol, 3-methyl-,acetate	123-92-2		*Muscodor albus* strain GBA		[71]
34	Cyclohexane,1-methyl-4-methylene	2808-80-2		*Muscodor albus* strain GBA		[69]
35	2,3-Dimethyl-3-isopropyl-cyclopentene	73331-73-4		*Muscodor albus* strain GBA		[69]
36	1-Butanol, 3-methyl	123-51-3		*Muscodor albus* strain GBA		[69]
37	Pyrrolidine	123-75-1		*Muscodor albus* strain GBA		[72]
38	Germacrene B	15423-57-1		*Muscodor albus* strain GBA		[72]
39	α-Sinensal	17909-77-2		*Muscodor albus* strain GBA		[69]
40	Propanoic acid, 2-methyl	79-31-2		*Muscodor albus* strain GBA		[73]
41	*Trans*-caryophyllene	87-44-5		*Muscodor albus* strain GBA	Anti-inflammatory agents	[73]
42	4-Piperidinone, 1-methyl	1445-73-4		*Muscodor albus* strain GBA		[73]
43	Acetic acid, 2-phenylethyl ester	103-45-7		*Muscodor albus* strain GBA		[73]
44	(+)-Vitrene	90250-82-1		*Muscodor albus* strain GBA		[73]

In recent years, some new ginkgo endophytes and secondary metabolites have been discovered. Guo et al. [20, 21] discovered a new amide compound from *Plantactinospora* sp. NEAU-gxj3, Cao et al. [22] found the metabolite sporothriolide from the *Nodulisporium* of *G. biloba*, which has anti-phytopathogenic activity.

## Application of secondary metabolites from *Ginkgo biloba*

Following the discovery by Schwabe of Germany that *Ginkgo biloba* contains active ingredients—ginkgo flavonoids and ginkgolides for the prevention and treatment of cardiovascular, cerebrovascular and neurological diseases, the researches about ginkgo has become more popular. Germany and France were the first countries in the world to develop ginkgo leaf products. In the mid-1970s, they first developed *Ginkgo biloba* leaves for the treatment of cardiovascular diseases. Since then, there are more than 50 kinds of ginkgo products on the market.

In the application, *Ginkgo* can be used with the extracts. Some examples, a substance EGb 761 extracted from *Ginkgo biloba* has shown to be effective against Noise-induced hearing loss (NIHL) in an animal model. This substance is assumed to protect the cochlea from hair cell loss after intensive noise exposure by reducing reactive oxygen species (ROS). Further effects of EGb 761 on the cellular and systemic levels of the nervous system make it a promising candidate not only for protection against NIHL but also for its secondary comorbidities like tinnitus [41]; One *Ginkgo biloba* extract (GbE) was used as a nontoxic natural reducing and stabilizing agent for preparing cytocompatible graphene. The as-prepared GbE-reduced graphene oxide (Gb-rGO) showed significant biocompatibility with cancer cells. Addition of GbE makes rGO producing procedure cost-effective and green. This method could be used for various biomedical applications, such as tissue engineering, drug delivery, biosensing, and molecular imaging [42].

Some application has been using a part of the plant. Another example, *Ginkgo* tea is a kind of health food produced from *Ginkgo biloba* leaves. Two kinds of glycosidase were used to improve the flavor of *Ginkgo* tea, and three kinds of bioactivities were selected to investigate the health care function of the tea infusion [43].

The *Ginkgo* preparation mainly includes capsules, tablets, granules, tea bags. Capsules and tablets are most popular in the formulation of the product. Recently, new preparation like shampoo, facial cleanser and hair moisturizer have been introduced in cosmetics applications. Most of the ginkgo products on the market are registered as health foods and a few are registered as over-the-counter drugs.

In many existing products, especially in the medicines, 24% of total flavonoids and 6% of ginkgolides are the basic quality requirements for *Ginkgo biloba* extracts. Some famous manufacturers proposed higher standards. They appended ginkgolides A, B, C, J and biloba lactone as the quality indicators and generally required the content of ginkgolides A, B, C, J greater than 2.5%, the content of biloba lactone greater than 2.6%.

On the basis of data about the endophytes and secondary metabolites in *G. biloba*, the catalogue is diverse in terms of structural complexity and lots of them have promising biological activities, which have the potential to be a source of new pharmaceutical agents which have a constant, critical need to combat cancers, viral infections, infectious diseases, and autoimmune disorders. There is also a growing need to fight insect-borne diseases of both animals and plants as climatological changes provide conditions conducive to more intensive outbreaks of these events. The fight against any disease is a dynamic equilibrium between advances in chemotherapy and natural selection in infectious or invasive agents. If the scientific community is to maintain parity in this never-ending struggle, then new sources of novel, bioactive chemotherapeutic agents must be found.

It appears that the mechanism by which endophytes produce secondary metabolites that mimic those produced by their host plants is far from clear. Even though efforts to unravel the pathway genes in the endophytes, it has failed to detect critical genes corresponding to those existing in plants, our understanding of the mechanisms associated with the development of different diseases increases, our ability to use this knowledge to select for ever more potent and selective compounds should increase commensurately. Endophytes of *G. biloba* will continue to provide a fertile arena for these quests.

## Prospects

With human aging process is accelerating, it has been common pursuit for a healthy and high-quality living. Since *Ginkgo biloba* preparations have a worldwide reputation as natural medicines and healthy products, *Ginkgo* development and the prospects are attractive. In the United States, *Ginkgo biloba* extracts have been on the list of imported drugs. *Ginkgo* products on the market are almost all products of American companies, and few products have been seen in Europe. At present, the European market is basically occupied by French and German products. Most of the *Ginkgo* extracts on the US market are produced by Japan and South Korea, a small portion is purchased from China.

**Fig. 7:**
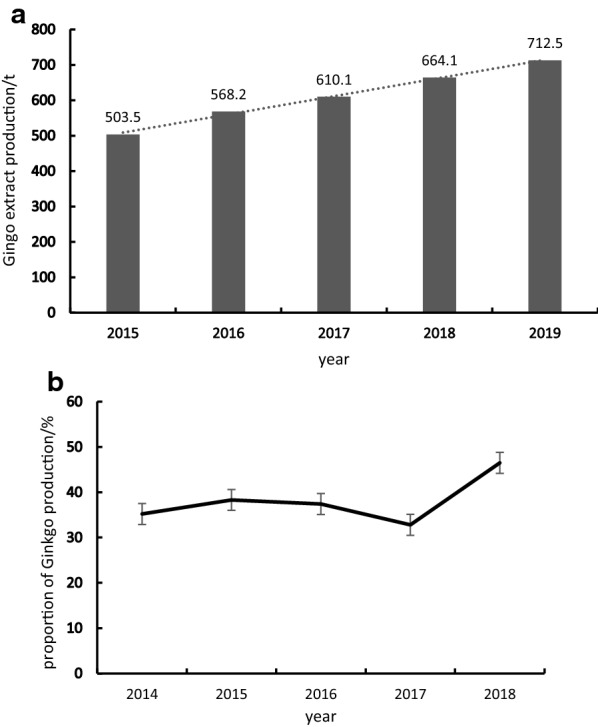
The production of Ginkgo extracts in China and its proportion in the world market. **a** The production of Ginkgo extracts in China from 2015 to 2019; **b** the proportion of China Ginkgo products in the world market from 2014 to 2018

Although comparing with the developed countries, China market is not competitive and too weak to take the risks, the potential of China’s *Ginkgo* development is still worth looking forward to. China is the birthplace and main producing area of the world’s *Ginkgo*. Many excellent *Ginkgo* germplasm resources are valuable treasures for China. With the sharp increase in *Ginkgo* resources and products output in China, the market has become more concerned at present (Fig. 7). At present, the *Ginkgo* products in China have low added-value and quality. In the development of ginkgo industry in China, it is necessary to increase the quality standardization and to improve the scientific research efforts and the production technology of *Ginkgo* preparations. It deserves to initiate new and technological products on flavonoids, bilobalide, polyisoprene, etc. Especially some new application in other industries should be explored, such as supplying in cytocompatible graphene preparation.

Chinese people have a tradition to have *Ginkgo* preparation as healthy products. China’s population accounts for about a quarter of the world’s total population. Therefore, the *Ginkgo* products in China should have more concerns on the domestic market and at the same time expand the international market with high-quality and featured products.

## Data Availability

Not applicable.

## Abbreviations

*G. biloba*: *Ginkgo biloba*
CD: electronic circular dichroism
Gb-rGO: gbE-reduced graphene oxide
NIHL: noise-induced hearing loss
ROS: reactive oxygen species
